# Linear associations of triglyceride-glucose body mass index and atherogenic index of plasma with the risk of diabetes: a retrospective cohort study

**DOI:** 10.3389/fendo.2026.1812819

**Published:** 2026-04-17

**Authors:** Sha Wang, Kui Li, Xi Wang, Lingjun Zhou, Chancui Deng, Guanxue Xu

**Affiliations:** 1 Department of Cardiovascular Medicine, Affiliated Hospital of Zunyi Medical University, Zunyi, Guizhou, China; 2 Department of Cardiovascular Medicine, The Fifth Affiliated Hospital of Zunyi Medical University (Zhuhai), Zhuhai, Guangdong, China

**Keywords:** atherogenic index of plasma, diabetes mellitus, insulin resistance, retrospective study, triglyceride glucose-body mass index

## Abstract

**Background:**

This research intends to examine the relationship between triglyceride glucose-body mass index (TyG-BMI) and the Atherogenic Index of Plasma (AIP) in relation to the risk of diabetes, as well as evaluate their predictive capabilities.

**Methods:**

This retrospective cohort study included data from 116,662 Chinese adults with normal baseline glucose levels. Participants were categorized according to tertiles of triglyceride-glucose body mass index (TyG-BMI; TT1-TT3) and atherogenic index of plasma (AIP; TA1-TA3), as well as nine combined categories (TC1-TC9). Incident diabetes mellitus (DM) was assessed after a mean follow-up of 37.4 months, and the primary endpoint was new-onset DM.

**Results:**

The overall incidence of DM was 2.3% (n = 2,681). After adjustment for covariates, both TyG-BMI and AIP were independent risk factors for DM (TyG-BMI: OR = 1.0100, *p* < 0.001; AIP: OR = 2.7800, *p* < 0.001). In combined analyses, participants in the highest combined category (TC9) had a 15.02-fold higher DM risk than those in the lowest combined category (TC1) in Model 1 and a 3.06-fold higher risk after multivariable adjustment in Model 2. Restricted cubic spline analyses demonstrated dose-response associations. The observed inflection points were 296.292 for TyG-BMI and 0.677 for AIP within this cohort, and addition of TyG-BMI, AIP, or their combination significantly improved DM risk prediction (all *p* < 0.001).

**Conclusions:**

In this Chinese adult cohort, TyG-BMI and AIP were significantly associated with incident DM and showed added predictive value. Incorporating TyG-BMI, AIP, or their combination into the baseline risk model significantly improved diabetes risk prediction.

## Background

As one of the world’s most prevalent chronic diseases, diabetes mellitus (DM) has become a major public health challenge.In recent years, the prevalence of DM has risen in both developed and developing countries (1), imposing a significant burden on public health and healthcare systems (2). The latest statistics indicate that in 2021, 529 million people worldwide were affected by DM, and this figure is projected to rise to approximately 1.31 billion by 2050 (2). Notably, China has the world’s largest diabetes population, totaling 140 million in 2021 (3). In addition, the associated complications impose a significant health and social burden. However, diabetes is largely preventable and can even be reversed in some cases if detected and managed early. Therefore, identifying an easily accessible biomarker in clinical practice could aid in primary prevention and early detection of individuals at risk for diabetes, thereby supporting the development of effective prevention strategies.

Insulin resistance (IR) is a key pathophysiological factor in type 2 diabetes(T2D). The current gold standard for measuring IR, the hyperinsulinemic-euglycemic clamp (HEC) (4) is time-consuming, costly, and challenging to implement in clinical practice. Therefore, a more convenient approach is needed to predict diabetes. Recently, several new indices have been proposed to predict the onset of diabetes, including the visceral adiposity index (VAI) (5) and the triglyceride–glucose index (TyG) (6). The triglyceride–glucose–body mass index (TyG–BMI) is a newly developed, reliable surrogate for IR, comprising three readily measurable parameters: body mass index (BMI), triglycerides (TG), and fasting plasma glucose (FBG).Recent epidemiological studies indicate that TyG–BMI is highly effective in identifying IR and valuable for predicting diabetes and other chronic conditions (4, 7–9). Although earlier research has examined the linear association between TyG–BMI and new-onset diabetes, the optimal TyG–BMI range in the general population is still unclear. Atherogenic Index of Plasma (AIP), proposed by Dobiásová and Frohlich in 2001 (10), is calculated as the logarithmic conversion of the total cholesterol (TG) to high-density lipoprotein cholesterol (HDL-C) ratio (11, 12). Previous studies suggest that individuals with dyslipidemia are at an elevated risk of progressing to end-stage DM (13). AIP was initially introduced as a novel biomarker of plasma atherosclerosis for predicting cardiovascular disease risk. Numerous recent studies have confirmed the link between AIP and the risk of IR–related metabolic disorders, including obesity (14, 15), prediabetes (16), DM (17, 18), and metabolic syndrome (19). Recent evidence also indicates that AIP is closely linked to the incidence of prediabetes or DM. However, these correlation studies remain limited, and their effect sizes may differ across ethnic groups (12, 18–20). Therefore, we performed a nationally representative *post hoc* analysis using data from a computerized database created by the Fortune Health Group in China, encompassing medical records of individuals who underwent health examinations between 2010 and 2016. Our primary objective was to investigate the associations of the TyG–BMI index and AIP with DM risk.

## Methods

### Study design and population

#### Data source and study participants

This study drew its data from the Dryad public database (https://datadryad.org/stash/dataset/doi:10.5061%2Fdryad.ft8750v), originally provided by Chen et al. (21). The dataset comprises medical data from 211,833 individuals who underwent health examinations at the Rich Healthcare Group across 32 sites in 11 Chinese cities between 2010 and 2016.

**Figure 1:**
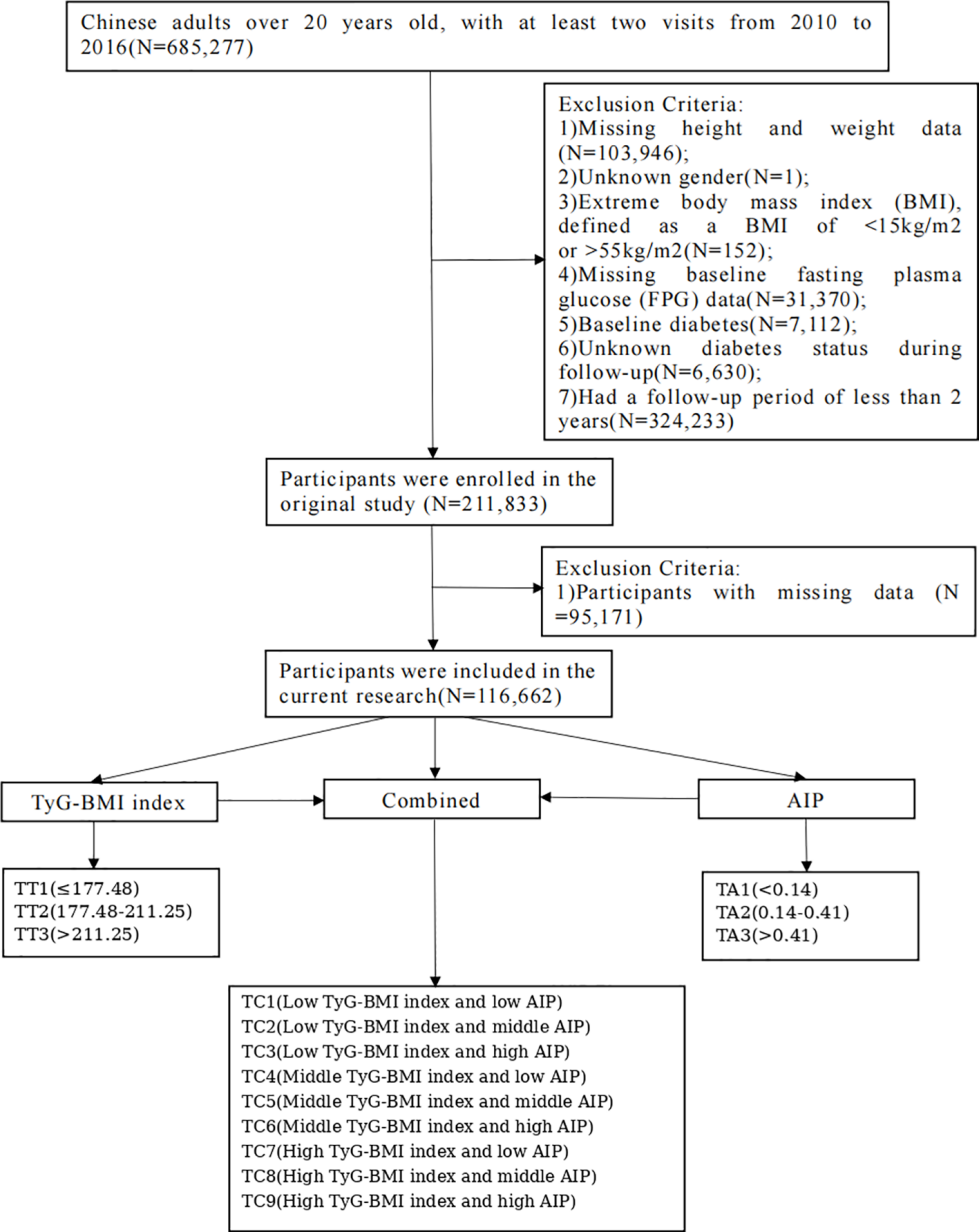
Flow chart of enrolled participants. TyG-BMI, triglyceride-glucose body mass index; AIP, atherogenic index of plasma. According to TyG-BMI tertiles, participants were categorized as TT1 (≤177.48), TT2 (177.48-211.25), and TT3 (>211.25). According to AIP tertiles, participants were categorized as TA1 (<0.14), TA2 (0.14-0.41), and TA3 (>0.41). Based on the combination of TyG-BMI and AIP tertiles, participants were further categorized into nine joint groups: TC1 = TT1 + TA1, TC2 = TT1 + TA2, TC3 = TT1 + TA3, TC4 = TT2 + TA1, TC5 = TT2 + TA2, TC6 = TT2 + TA3, TC7 = TT3 + TA1, TC8 = TT3 + TA2, and TC9 = TT3 + TA3.

The original study recruited 685,277 Chinese adults aged >20 years who made at least two visits between 2010 and 2016. The exclusion criteria were: (1) missing height and weight data (n = 103,946); (2) unknown sex (n = 1); (3) extreme BMI (<15 kg/m² or >55 kg/m²; n = 152); (4) missing baseline FBG data (n = 31,370); (5) baseline diabetes (n = 7,112); (6) unknown diabetes status during follow-up (n = 6,630); and (7) follow-up <2 years (n = 324,233). Ultimately, 211,833 participants were included in the original study. The present study further explored the association of TyG-BMI and AIP with DM risk. According to additional ADA diagnostic criteria, participants missing required data (n = 95,171) were excluded, leaving 116,662 participants for the current analysis (**Figure 1**). The study received an ethics waiver from the Committee of the Affiliated Hospital of Zunyi Medical University (Approval No. [KLLY-2024-171]) and met Dryad’s publication criteria.

Because this was a retrospective secondary analysis of de-identified data from a public database, blinding was not applicable.

### Data measurement and definition

The researchers collected sociodemographic data from participants using a standardized questionnaire, including age, sex, smoking status, drinking status, and family history of diabetes. Blood pressure, height, and weight were measured by trained medical staff using standardized examination procedures, and blood pressure was measured with a mercury sphygmomanometer. Smoking and drinking status were categorized into four groups at baseline: never, ever, current, and not recorded. Weight was measured with participants wearing light clothing and no shoes. BMI was calculated as weight (kg) divided by height squared (m²).

Fasting venous blood samples were collected from participants by healthcare professionals after at least 10 hours of fasting. Lipids, glucose, low-density lipoprotein cholesterol (LDL-C), HDL-C, alanine aminotransferase (ALT), aspartate aminotransferase (AST), FBG, serum creatinine (Scr), and blood urea nitrogen (BUN) levels were measured using an automated analyzer (Beckman Coulter AU5800, Brea, CA, USA). Scr and BUN levels, as well as FBG levels, were measured using the glucose oxidase method.

The TyG-BMI has been identified as a reliable indicator of IR, reflecting the metabolic health of different populations. AIP is a reliable marker for assessing atherosclerosis and serious cardiovascular events, calculated as the logarithm of the ratio of TG to HDL-C. A strong correlation has been found between AIP and T2DM, with higher AIP levels associated with an increased risk of developing T2DM. The TyG–BMI index is defined as ln[TG (mg/dL) × FBG (mg/dL)/2] × BMI (22). The AIP is calculated as lg (TG/HDL-C) (12). eGFR was calculated according to the MDRD formula (23):eGFR = 186 × Cr-^1.154^ ×age^0.203^ in men, and eGFR = 186 × Cr-^1.154^ ×age^0.203^ × 0.742 in women.DM was defined as a fasting blood glucose level of ≥7.00 mmol/L and/or self-reported diabetes. Patients were reviewed on the date of diagnosis or at the last visit, whichever came first (24).

### Statistics analysis

Continuous variables were tested for normality using the Shapiro-Wilk test. Normally distributed variables are presented as mean ± standard deviation (SD), whereas non-normally distributed variables are presented as median (interquartile range). Categorical variables are presented as counts and percentages. Comparisons between two groups were performed using the t test for normally distributed continuous variables and the rank-sum test for non-normally distributed variables or variables with heteroscedasticity. Comparisons among three groups were performed using one-way analysis of variance (ANOVA) for normally distributed variables with homogeneity of variance and the rank-sum test otherwise. The chi-square test was used for categorical variables. Missing continuous variables, including ALT, AST, serum creatinine (Scr), systolic blood pressure (SBP), and diastolic blood pressure (DBP), were handled using appropriate estimates of the mean or median. Missing smoking and drinking data were retained as the category “not recorded”. Logistic regression analyses were used to evaluate the associations of TyG-BMI and AIP with DM risk, and odds ratios (ORs) with 95% confidence intervals (CIs) were calculated.

In this research, the initial analysis, referred to as Model 1, was conducted without any adjustments. Model 2 used a multivariable approach. Before developing the multivariable model, covariance among the TyG-BMI index, the AIP index, and other covariates was assessed by calculating the generalized variance inflation factor (GVIF), where significant covariance among covariates was identified when the GVIF raised to the power of 1/(2Df) was ≥2. The least absolute shrinkage and selection operator (LASSO) regression was then used to select covariates from the initial variable set. For the multivariable logistic regression analysis in the overall study population, the adjusted covariates included age, BMI, systolic blood pressure (SBP), diastolic blood pressure (DBP), estimated glomerular filtration rate (eGFR),FBG, smoking status, family history of diabetes, and sex. In addition, restricted cubic spline (RCS) analyses were performed to further explore the associations of TyG-BMI and AIP with DM risk. Because the associations were approximately linear on either side of the internal spline-derived inflection points, linear models were used to estimate the OR associated with each standard deviation (SD) increase in TyG-BMI and AIP.

Receiver operating characteristic (ROC) curve analysis was performed to evaluate model discrimination, and the area under the curve (AUC) was calculated. Differences in AUCs were compared using the DeLong test. Subgroup analyses were conducted according to age and sex using logistic regression. A two-sided *p* < 0.05 was considered statistically significant. All statistical analyses were performed using R software (version 4.2.3, R Foundation for Statistical Computing, Vienna, Austria).

## Results

### Baseline characteristics of participants

In this study, the median age of the 116,662 participants was 41 years (interquartile range, 34–53 years). Males accounted for 53.8% of the overall study population, and 1,888 of the 2,681 incident DM cases (70.4%) occurred in men during a mean follow-up of 37.4 months. The baseline characteristics of the participants are presented in **Table 1**. Participants who developed DM had significantly higher BMI, DBP, SBP, TC, TG, LDL-C, FBG, ALT, AST, BUN, Cr, AIP, and TyG-BMI values, as well as lower HDL-C and eGFR values, than those who did not develop DM (all *p* < 0.05). Baseline characteristics stratified by TyG-BMI and AIP are shown in **Supplementary Tables 1**, **S2**.

**Table 1:** Demographic and clinical baseline data for the two groups.

Variables	Total N=116,662	DM patients N=2,681	non-DM patients N=113,981	*p*
Age (years)	41 (34,53)	57 (47,65)	41 (34,52)	<0.001
Male	62,759 (53.8)	1,888 (70.4)	60,871 (53.4)	<0.001
BMI (kg/m^2^)	23.10 (21.00,25.400)	25.90 (23.70,28.00)	23.10 (20.90,25.30)	<0.001
SBP (mmHg)	118 (107,130)	131 (119,143)	118 (107,129)	<0.001
DBP (mmHg)	73 (66,81)	80 (72,88)	73 (66,81)	<0.001
Smoking				<0.001
Current	6,658 (5.7)	257 (9.6)	6,401 (5.6)	
Former	1,326 (1.1)	46 (1.7)	1,280 (1.1)	
Never	24,649 (21.1)	389 (14.5)	24,260 (21.3)	
Not recorded	84,029 (72.0)	1,989 (74.2)	82,040 (72.0)	
Drinking				0.002
Current	872 (0.7)	31 (1.2)	841 (0.7)	
Former	5,524 (4.7)	116 (4.3)	5,408 (4.7)	
Never	26,237 (22.5)	545 (20.3)	25,692 (22.5)	
Not recorded	84,029 (72.0)	1,989 (74.2)	82,040 (72.0)	
Family history	2,634 (2.3)	98 (3.7)	2,536 (2.2)	<0.001
TG (mmol/L)	1.10 (0.76,1.66)	1.70 (1.17,2.50)	1.10 (0.76,1.64)	<0.001
TC (mmol/L)	4.70 (4.16,5.32)	5.00 (4.40,5.67)	4.70 (4.15,5.31)	<0.001
HDL-C (mmol/L)	1.35 (1.16,1.56)	1.27 (1.08,1.49)	1.35 (1.16,1.56)	<0.001
LDL-C (mmol/L)	2.70 (2.29,3.16)	2.86 (2.39,3.34)	2.70 (2.29,3.16)	<0.001
FBG (mmol/L)	4.9 (4.6,5.3)	6.0 (5.5,6.5)	4.9 (4.6,5.3)	<0.001
ALT (U/L)	18 (13,28)	25 (18,40)	18 (13,27)	<0.001
AST (U/L)	22 (19,27)	25 (21,31)	22 (19,27)	<0.001
BUN (mmol/L)	4.570 (3.84,5.40)	4.89 (4.10,5.76)	4.56 (3.84,5.39)	<0.001
Scr (µmol/L)	70 (58,81)	73 (62,82)	69 (58,81)	<0.001
eGFR (mL/min/1.73 m2)	102 (90,115)	97 (85,111)	102 (90,115)	<0.001
TyG-BMI index	193.90 (169.21,221.19)	233.22 (210.22,258.05)	193.06 (168.76,219.92)	<0.001
AIP	0.27 (0.08,0.49)	0.49 (0.30,0.70)	0.27 (0.07,0.48)	<0.001

Data are presented as mean ± SDs, medians (interquartile ranges), or n (%).

### Clinical outcomes

The overall occurrence of DM was found to be 2.3% (N = 2,681). Both univariate (OR = 1.0200; 95% CI = 1.0200-1.0200; *p* < 0.001) and multivariate (OR = 1.0100; 95% CI = 1.0100-1.0100; *p* < 0.001) logistic regression analyses indicated a significant association between the TyG-BMI and the likelihood of developing DM (see **Table 2**). In Model 2, when adjusting for various potential risk factors, the incidence of DM was 1.58 times greater in the TT2 group in comparison to the TT1 group (OR = 1.5800; 95% CI = 1.3000-1.9000; *p* < 0.001), and the TT3 group exhibited a 3.05 times higher incidence than the TT1 group (OR = 3.0500; 95% CI = 2.5400-3.6500; *p* < 0.001). Both univariate and multivariate logistic regression analyses showed that AIP was significantly associated with the risk of developing DM (see **Table 3**). In Model 2, compared with the TA1 group, the TA2 group had a 1.43-fold higher risk (OR = 1.4300; 95% CI = 1.2300-1.6600; p < 0.001), and the TA3 group had a 2.12-fold higher risk (OR = 2.1200; 95% CI = 1.8300-2.4700; p < 0.001). Logistic regression analysis showed that the incidence of DM in the TC9 group was significantly higher than that in the TC1 group in both models (see **Table 4**). In Model 2, the TC9 group had a 3.06-fold higher risk than the TC1 group (OR = 3.0600; 95% CI = 2.4400-3.8200; p < 0.001).

**Table 2:** Association between TyG-BMI tertiles and incident DM.

Variables	Events/N	Model 1			Model 2		
		OR	95% CI	*p*	OR	95% CI	*p*
TyG-BMI index	2,681/116,662	1.02	1.02-1.02	<0.001	1.01	1.01-1.01	<0.001
TT1	152/38,887	Reference			Reference		
TT2	548/38,887	3.64	3.04-4.36	<0.001	1.58	1.30-1.90	<0.001
TT3	1,981/38,888	13.68	11.59-16.14	<0.001	3.05	2.54-3.65	<0.001
*p* for trend				<0.001			<0.001

OR, Odds ratio; CI, confidence interval; TyG-BMI, triglyceride-glucose body mass index. TT1, TyG-BMI ≤177.48; TT2, 177.48 < TyG-BMI ≤211.25; TT3, TyG-BMI >211.25.

**Table 3:** Association between AIP tertiles and incident DM.

Variables	Events/N	Model 1			Model 2		
		OR	95% CI	*p*	OR	95% CI	*p*
AIP	2,681/116,662	8.87	7.88-9.99	<0.001	2.78	2.35-3.30	<0.001
TA1	294/38,891	Reference			Reference		
TA2	717/38,887	2.47	2.15-2.83	<0.001	1.43	1.23-1.66	<0.001
TA3	1,670/38,884	5.89	5.20-6.67	<0.001	2.12	1.83-2.47	<0.001
*p* for trend				<0.001			<0.001

OR, Odds ratio; CI, confidence interval; AIP, atherogenic index of plasma. TA1, AIP <0.14; TA2, 0.14-0.41; TA3, AIP >0.41.

Univariate (OR = 8.8700; 95% CI = 7.8800-9.9900; *p* < 0.001) and multivariate (OR = 2.7800; 95% CI = 2.3500-3.3000; *p* < 0.001) logistic regression analyses showed that AIP was significantly associated with the risk of developing DM (**Table 3**). In Model 2, after adjusting for potential risk factors, the incidence of DM was 1.43 times higher in the TA2 group compared to the TA1 group (OR = 1.4300; 95% CI = 1.2300-1.6600; *p* < 0.001), and 2.12 times higher in the TA3 group compared to the TA1 group (OR = 2.1200; 95% CI = 1.8300-2.4700; *p* < 0.001).

**Table 4:** Joint association of TyG-BMI and AIP tertiles with incident DM.

Variables	Events/N	Model 1			Model 2		
		OR	95% CI	*p*	OR	95% CI	*p*
TC1	103/26,752	Reference			Reference		
TC2	40/10,597	0.98	0.68-1.41	0.915	0.75	0.52-1.10	0.139
TC3	9/1,538	1.52	0.77-3.02	0.228	1.01	0.50-2.05	0.974
TC4	121/10,319	3.07	2.36-4.00	<0.001	1.21	0.92-1.60	0.168
TC5	247/18,214	3.56	2.82-4.48	<0.001	1.48	1.16-1.89	0.001
TC6	180/10,354	4.58	3.59-5.84	<0.001	1.72	1.32-2.24	<0.001
TC7	70/1,820	10.35	7.61-14.07	<0.001	2.35	1.68-3.27	<0.001
TC8	430/10,076	11.53	9.29-14.32	<0.001	2.59	2.05-3.26	<0.001
TC9	1,481/26,992	15.02	12.29-18.35	<0.001	3.06	2.44-3.82	<0.001
*p* for trend				<0.001			<0.001

OR, Odds ratio; CI confidence interval; TyG-BMI, triglyceride-glucose body mass index; AIP, atherogenic index of plasma. TC1-TC9 represent the nine combinations of TT1-TT3 and TA1-TA3.

Logistic regression analysis showed that the incidence of DM in the TC9 category (high TyG-BMI and high AIP) was 15.02 times higher than that in the TC1 category (low TyG-BMI and low AIP) in Model 1 (OR = 15.0200; 95% CI = 12.2900-18.3500; *p* < 0.001). In Model 2, after adjustment for potential confounders, the incidence of DM in the TC9 group remained 3.06 times higher than that in the TC1 group (OR = 3.0600; 95% CI = 2.4400-3.8200; *p* < 0.001).

**Figure 2:**
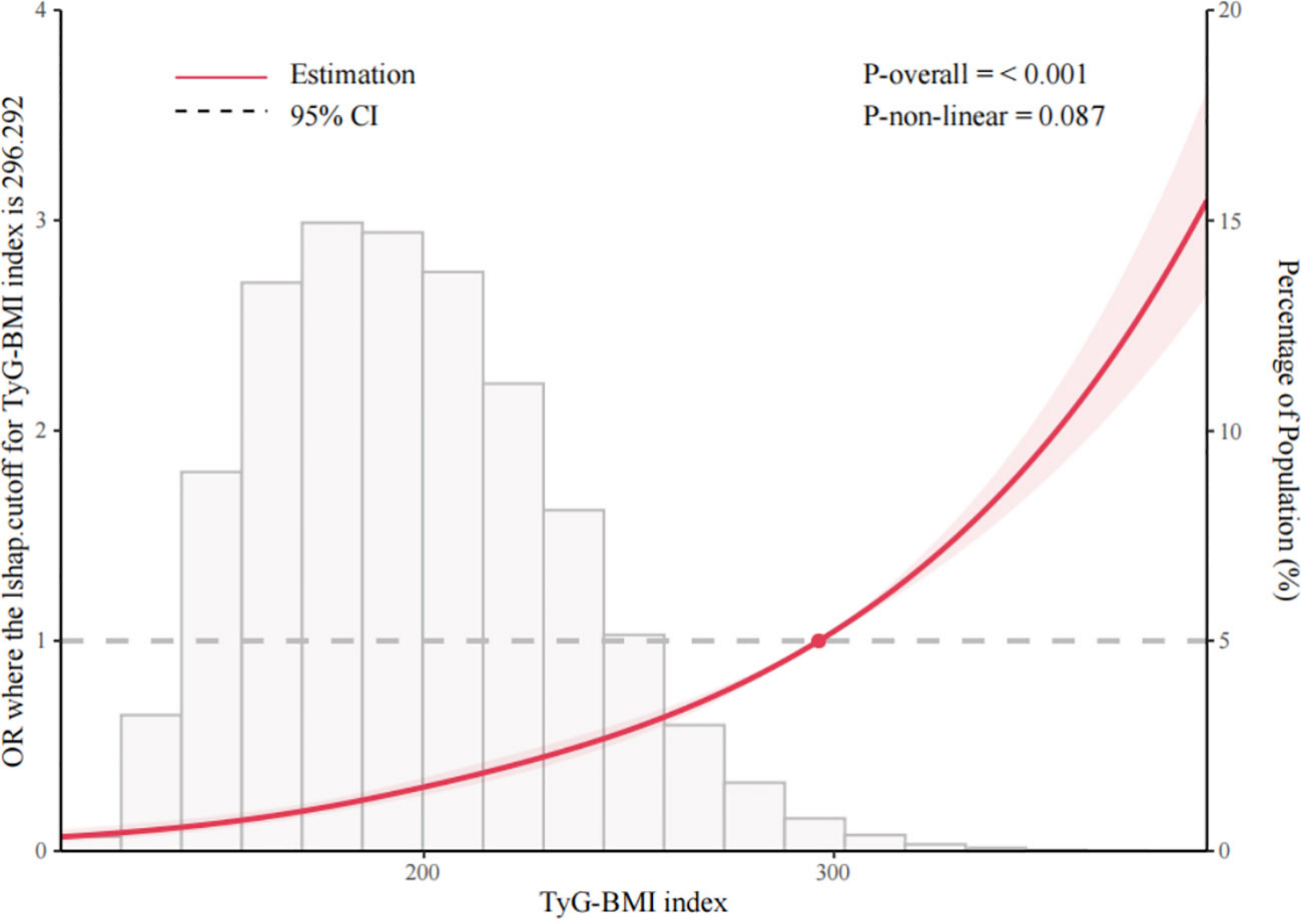
Linear association between triglyceride-glucose body mass index (TyG-BMI) and risk of diabetes mellitus (DM) in Chinese adults. Only 95% of the data are displayed. Odds ratios are shown by the solid line, and 95% confidence intervals (CIs) are shown by the shaded area.

**Figure 3:**
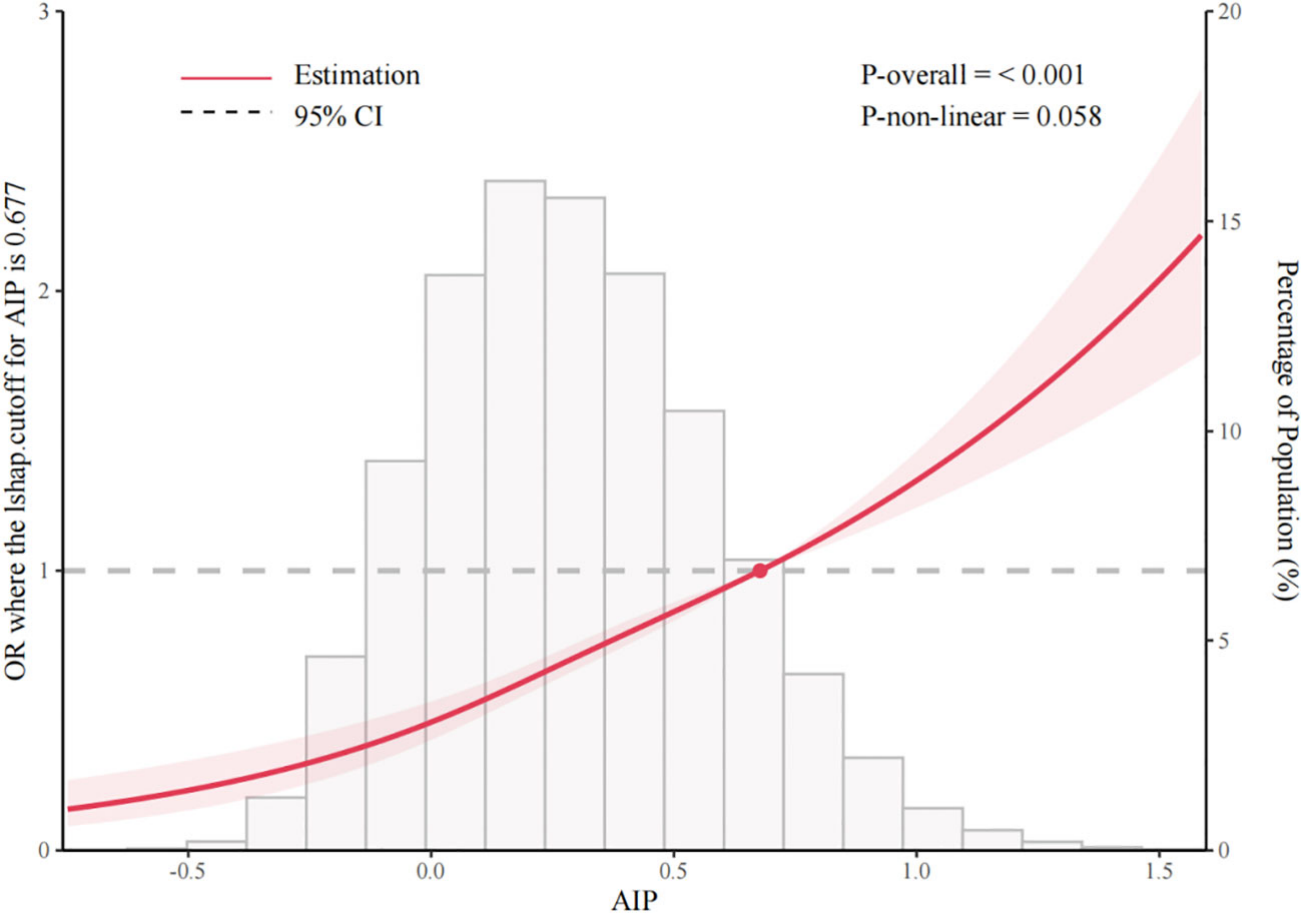
Linear association between atherogenic index of plasma (AIP) and risk of diabetes mellitus (DM) in Chinese adults. Only 95% of the data are displayed. Odds ratios are shown by the solid line, and 95% confidence intervals (CIs) are shown by the shaded area.

The RCS analysis showed a dose-response association between TyG-BMI and incident DM (nonlinearity *p* = 0.087) after adjustment in Model 2 (**Figure 2**). The cohort-derived inflection point was 296.292 for TyG-BMI. Above this value, the OR for predicted DM per standard deviation (SD) increase was 1.36 (95% CI: 1.15-1.61), whereas below this value it was 1.63 (95% CI: 1.55-1.72) (**Supplementary Table 3**). A similar dose-response association was observed between AIP and incident DM (nonlinearity *p* = 0.058) (**Figure 3**). The cohort-derived inflection point was 0.677 for AIP. At or above this value, the OR for predicted DM per SD increase was 1.07 (95% CI: 0.99-1.16), whereas below this value it was 1.36 (95% CI: 1.27-1.45) (**Supplementary Table 4**). These values should be interpreted as internal spline-derived inflection points rather than universal clinical cutoffs or normal reference ranges.

### Subgroups and sensitivity analysis

**Figure 4:**
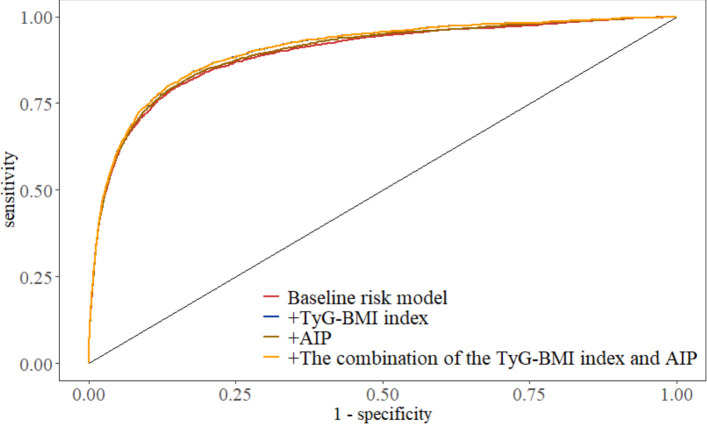
Receiver operating characteristic curves for assessing the predictive ability of triglyceride-glucose body mass index (TyG-BMI), atherogenic index of plasma (AIP), and their combination for diabetes mellitus (DM) risk. The baseline risk model included age, body mass index (BMI), systolic blood pressure (SBP), diastolic blood pressure (DBP), estimated glomerular filtration rate (eGFR), fasting blood glucose (FBG), smoking status, family history of diabetes, and sex.

Subgroup analyses stratified by age and sex were performed to evaluate the robustness of the associations between TyG-BMI, AIP, and diabetes risk (**Supplementary Tables 5**-**8**). In the overall population, we constructed prediction models and plotted ROC curves (**Figure 4**) to evaluate their performance for diabetes prediction: a baseline risk model, a baseline model plus TyG‑BMI index, a baseline model plus AIP, and a baseline model plus both TyG‑BMI index and AIP. Compared with the baseline risk model (AUC=0.8917, 95% CI: 0.8847‑0.8987), adding TyG‑BMI index, adding AIP, and combining both indices significantly improved the predictive performance (AUC=0.9030, 0.8958, and 0.9030, respectively; all P<0.001) (**Table 5**).This paragraph is presented independently as a Result section and is not included in the subgroup analysis.

**Table 5:** Incremental predictive value of TyG-BMI, AIP, and their combination for DM prediction.

Model	AUC (95% Cl)	*p*
Baseline risk model	0.8917(0.8847-0.8987)	Ref
+TyG-BMI index	0.9030(0.8967-0.9094)	<0.001
+AIP	0.8958(0.8890-0.9025)	<0.001
+The combination of the TyG-BMI index and AIP	0.9030(0.8967-0.9094)	<0.001

AUC, area under the curve; CI, confidence interval; TyG-BMI, triglyceride-glucose body mass index; AIP, atherogenic index of plasma; FBG, fasting blood glucose.

## Discussion

This large retrospective cohort study of 116,662 Chinese adults without baseline diabetes investigated the associations of TyG-BMI and AIP with incident DM. The main findings were as follows: (1) both TyG-BMI and AIP were significantly associated with DM risk; (2) adding TyG-BMI and AIP to the baseline risk model improved predictive performance; and (3) both markers showed dose-response associations with incident DM. Taken together, these findings suggest that TyG-BMI and AIP may serve as accessible surrogate markers of metabolic dysfunction and may help identify individuals at higher risk of diabetes in clinical practice.

The TyG index is well-established as a dependable indicator for evaluating IR, showing notable sensitivity and specificity. Its ease of use, affordability, and broad accessibility have made it a common tool in clinical settings. The authors declare that they have no conflicts of interest (25). Research regarding the TyG indices indicates that the triglyceride glucose-waist circumference (TyG-WC) demonstrates the greatest predictive precision for short-term diabetes risk, spanning 2 to 6 years. Conversely, the triglyceride glucose-waist height ratio (TyG-WHtR) shows superior predictive accuracy along with more consistent predictive thresholds for mid- to long-term diabetes prevalence, which ranges from 6 to 12 years (4). The TyG-BMI index was initially introduced in 2016 and has been recommended as a potential alternative indicator for IR (22). Various cross-sectional (26, 27) and prospective (7, 9) studies have demonstrated a positive correlation between the TyG-BMI index and the risk of diabetes, with evidence suggesting that its ability to predict risk is greater than that of the TyG index used independently (8, 28). The relationship between levels of the TyG-BMI index and the likelihood of developing DM is thoroughly examined. Previous cohort studies have extensively illustrated its predictive capability for DM risk across various populations (7). Several previous studies have investigated the association between the TyG-BMI index and the risk of developing DM. Song et al. (9)recruited 15,464 Japanese individuals for a retrospective cohort study with type 2 diabetes mellitus (T2DM) as the primary endpoint and followed them for 13 years. The results showed that baseline TyG-BMI was positively correlated with the risk of developing T2DM in Japanese individuals with normal blood glucose levels. Additionally, in young adults, women, non-hypertensive individuals, and non-alcohol drinkers, this risk was significantly higher. Wang et al. (28) recruited 699 Chinese individuals for a long-term follow-up of 6 and 34 years, with the endpoint being the risk of developing diabetes. They specified that a higher TyG-BMI index showed better predictability than the TyG index and HOMA-IR in both the short term (6 years) and the long term (34 years). This finding supports the use of the TyG-BMI index as a potential tool for assessing the risk of T2D in clinical practice. Additionally, RCS analysis showed that an increased TyG-BMI index was linearly associated with an increased risk of T2D.This is in line with our study, where the relationship between the TyG-BMI index and the risk of DM was linearly correlated. Qiao et al. (29) recruited 1,917 middle-aged and elderly participants, with the endpoint being the incidence of diabetes. The incidence was correlated with a J-shaped curve through 3 years of follow-up. The study revealed that the TyG-BMI index was positively and nonlinearly associated with the risk of new-onset diabetes in middle-aged and elderly Chinese. In addition, the RCS curves for the relationship between the TyG-BMI index and DM risk in our study were linear, whereas Qiao et al. reported a J-shaped relationship between the TyG-BMI index and the incidence of DM (29). This discrepancy may be due to differences in sample sizes, and it is difficult to compare the accuracy of the findings between the two studies due to the lack of relevant research (29). Therefore, further studies are needed to explore the association between the TyG-BMI index and DM risk in large randomized controlled trials (RCTs).

Previous research has consistently highlighted that atherogenic dyslipidemia, which is marked by an increase in TG levels and a decrease in HDL-C levels, plays a significant role in environments prone to diabetes as well as in individuals diagnosed with diabetes. This dyslipidemic state is not just a common observation; it is a defining characteristic of metabolic dysfunction that contributes to the pathophysiology of diabetes (30). Abnormal levels of lipids in the body can trigger a cascade of negative biological responses, including inflammation, endoplasmic reticulum stress, and lipotoxicity. These detrimental effects tend to reach their peak during the development of IR, highlighting the intricate relationship between lipid metabolism and glucose homeostasis (31). The AIP has emerged as a valuable tool in assessing cardiovascular risk, as it has been shown to have a significant correlation with both the size and density of lipoprotein particles, as well as the peroxidation rate of lipoproteins. The AIP is specifically calculated using the levels of TG and HDL-C, making it a practical and reliable marker for evaluating the extent of plasma atherosclerosis (32). Beyond its conventional uses, current research is increasingly investigating the relationship between AIP levels and the risk of developing DM. A substantial body of research has been dedicated to understanding this correlation, including a study conducted by Li et al. (33) which involved a cohort of 12,352 pre-diabetic adults. This cross-sectional study revealed a complex, non-linear relationship between AIP levels and pre-diabetes risk. Similarly, Jiang et al. (34) conducted a study that included 12,060 participants who were transitioning from prediabetes to diabetes. Their findings indicated a positive nonlinear association between AIP levels and the risks associated with both prediabetes and T2DM. Further reinforcing this association, Yang et al. (35) investigated a group of 15,421 pre-diabetic individuals. The collective insights from these studies underscore the potential of the AIP as a significant biomarker not only for cardiovascular risk but also for the risk of diabetes, providing a compelling argument for its incorporation into preventive health strategies aimed at diabetic risk management. In this multicenter retrospective cohort study, which involved a median follow-up period of 2.9 years, the RCS analysis revealed a complex, non-linear relationship between the AIP and the progression from pre-diabetes to either normal fasting glucose (NFG) or diabetes. The research conducted by Yin et al. (20) included a substantial cohort of 9,245 participants, highlighting that there exists an inverse and non-linear L-shaped association between AIP and IR. Furthermore, a J-shaped relationship was identified between AIP levels and the incidence of T2D. Notably, the study indicated that the positive correlation between AIP and T2D was markedly more pronounced in female participants compared to their male counterparts. In agreement with these findings, our study similarly indicated that the relationship between AIP and the risk of developing DM was significantly stronger in women than in men, emphasizing the potential gender differences in metabolic responses to AIP. In an analysis involving diverse populations, Sun et al. (36) enlisted a total of 40,633 overweight and obese participants, some diagnosed with diabetes and others without. This extensive study concluded that AIP serves as an independent marker associated with T2DM specifically in individuals classified as overweight or obese, further supporting the notion of a non-linear association. Interestingly, our findings regarding the relationship between the AIP index and diabetes risk revealed a linear pattern, in contrast to the conclusions drawn by Yang et al., who employed the same dataset as ours but asserted the presence of a non-linear relationship (35). The discrepancies between these findings may stem from variations in the glycemic status of the study participants and the differences in sample sizes across the cohorts analyzed. Moreover, the lack of comprehensive research in this field complicates the comparison of accuracy regarding these findings, underscoring the need for further investigation to elucidate the underlying mechanisms and relationships at play (35). Consequently, it is important to recognize that the connection between AIP and glycemic status is likely to differ among various populations. This variability underscores the need for a more comprehensive understanding of how different demographic and health factors may influence this relationship. Nonetheless, the current hypothesis remains unverified, and we look forward to future research that may yield more robust and conclusive evidence to clarify these associations.

To our knowledge, this is the first study to combine TyG-BMI and AIP to assess DM risk. Participants in the highest combined category (TC9) had a substantially higher risk of DM than those in the lowest category (TC1), and the combined marker improved discrimination beyond the baseline model. The spline-derived inflection points (TyG-BMI 296.292 and AIP 0.677) should be regarded as cohort-specific internal thresholds rather than established clinical cutoffs, and external validation is required before clinical use. A plausible biological explanation for the synergistic effect of TyG-BMI and AIP is that they capture overlapping but non-identical components of metabolic dysfunction. TyG-BMI integrates fasting blood glucose, triglycerides, and adiposity, and therefore reflects insulin resistance, glycemic burden, and obesity-related metabolic stress. In contrast, AIP, calculated from TG and HDL-C, reflects atherogenic dyslipidemia and an adverse lipoprotein profile. Excess adiposity increases free fatty acid flux to the liver, promotes hepatic very-low-density lipoprotein production, and worsens insulin resistance. At the same time, hypertriglyceridemia and reduced HDL-C may aggravate lipotoxicity, oxidative stress, chronic low-grade inflammation, and beta-cell dysfunction, thereby further impairing glucose homeostasis. These interacting pathways may explain why combining TyG-BMI and AIP provided better risk stratification than either index alone. Nevertheless, the molecular mechanisms remain incompletely understood and warrant further study.

Additional RCS analysis indicated that this linear association showed a dose-response effect. Furthermore, ROC curves illustrated that the combination of the TyG-BMI index and AIP produced a greater AUC for predicting DM risk than AIP on its own, similarly to the TyG-BMI index alone. We propose that this observation may be due to the distinct impacts exerted by the TyG index and AIP. The TyG index primarily evaluates the level of IR and is composed of three easily quantifiable parameters: BMI, TG, and FBG. Conversely, AIP serves as a dependable indicator for evaluating atherosclerosis and severe cardiovascular incidents, derived from the logarithmic ratio of TG to HDL-C. Through the comparison of the area under the ROC curve, we contend that our FBG levels significantly influence the outcomes. This investigation identified a robust link between AIP and T2DM, noting that increased AIP levels correlate with a heightened risk of developing T2DM (18). Nevertheless, due to our inclusion of adults whose fasting glucose levels were below 7.0 mmol/L, we did not categorize patients with prediabetes. Furthermore, in light of the fact that no prior research has integrated the TyG-BMI index with AIP, we could not verify the predictive benefits of this combination in a different cohort. This observation is anticipated to be corroborated in various populations. In future investigations, larger prospective cohort studies are necessary to ascertain the thresholds for the TyG-BMI index/AIP and to examine their predictive significance for diabetes management. In the analyses of subgroups, the interaction p-values concerning the TyG-BMI index and AIP by gender all exceeded 0.05, whereas the interaction p-values related to age were consistently below 0.01. The variations witnessed in subgroup analyses could be attributed to differences among individuals or sample sizes. However, the mechanisms that may underpin the dose-response relationship between the TyG-BMI index, AIP, and diabetes risk remain vague and may involve several factors. Despite the ambiguity surrounding the molecular and biological pathways that connect the TyG-BMI index and AIP to diabetes, there exists a potential link to IR. IR plays a critical role in both the initiation and advancement of diabetes (37), Elevated levels of glucose can result in heightened production of reactive oxygen species, which are detrimental to pancreatic β-cells (38). TG levels may play a role in IR (39), and an abundance of TG within pancreatic cells can impair β-cell functionality (38). Moreover, rising triglyceride levels result in a surge of free fatty acids (FFA), which can induce changes in insulin signaling in pancreatic α-cells and enhance the secretion of glucagon in a hyperglycemic state, potentially leading to IR (40). Conversely, IR can hinder the lipolysis of TG, which in turn elevates FFA concentrations in the liver while aggravating TG levels by lowering HDL-C due to a decrease in ApoA-I expression, a protein essential for HDL-C production (41). BMI is often utilized to evaluate the risk of obesity and various metabolic disorders, and it has been linked with a higher likelihood of developing DM (42). The possible mechanism connecting the TyG-BMI index and the AIP to diabetes risk could involve the interactions between FBG, TG, and BMI in relation to IR.

### Strength and limitations

To the best of our knowledge, this is the first study to jointly evaluate TyG-BMI and AIP in relation to incident DM using logistic regression, ROC analysis, and restricted cubic spline modeling in a large Chinese cohort. The large sample size and standardized health examination data strengthen the statistical precision of the findings. However, several limitations should be acknowledged. First, this was a retrospective secondary analysis, and residual confounding from unmeasured variables cannot be excluded. Second, a substantial number of participants were excluded because of follow-up shorter than 2 years, which may have introduced selection bias and may limit generalizability. Third, the cohort comprised Chinese adults, so the findings may not be directly generalizable to other ethnic populations. Fourth, the database did not allow detailed characterization of prediabetes or inclusion of oral glucose tolerance test or glycated hemoglobin data, which may have led to outcome misclassification in some individuals. Fifth, the spline-derived inflection points were obtained internally and require external validation before clinical application. Future prospective multicenter studies should validate these findings in diverse populations, examine longitudinal changes in TyG-BMI and AIP, and compare their performance with other insulin resistance indices.

## Conclusion

In this retrospective cohort of Chinese adults, both TyG-BMI and AIP were independently and linearly associated with incident DM, and their combination improved risk prediction beyond a baseline clinical model. These findings support the potential value of combining markers of glucose-lipid metabolism and adiposity for diabetes risk stratification. Prospective multicenter studies are needed to validate the observed inflection points and clarify the biological mechanisms underlying these associations.

## Data Availability

The original contributions presented in the study are included in the article/**Supplementary Material**. Further inquiries can be directed to the corresponding authors.

## Glossary

AIP: atherogenic index of plasma
ALT: alanine aminotransferase
AST: aspartate aminotransferase
AUC: area under the curve
BMI: body mass index
BUN: blood urea nitrogen
CI: confidence interval
Cr: creatinine
DBP: diastolic blood pressure
DM: diabetes mellitus
eGFR: estimated glomerular filtration rate
FBG: fasting blood glucose
GVIF: generalized variance inflation factor
HDL-C: high-density lipoprotein cholesterol
HEC: hyperinsulinemic-euglycemic clamp
HOMA: homeostatic model assessment
HOMA-IR: homeostatic model assessment of insulin resistance
IR: insulin resistance
LDL-C: low-density lipoprotein cholesterol
NFG: normal fasting glucose
OR: odds ratio
RCS: restricted cubic spline
ROC: receiver operating characteristic
SBP: systolic blood pressure
TC: total cholesterol
TG: triglycerides
TyG-BMI: triglyceride-glucose body mass index
TyG-WC: triglyceride-glucose waist circumference
TyG-WHtR: triglyceride-glucose waist-height ratio
VAI: visceral adiposity index.
