# Supplementary material for: Linear associations of triglyceride-glucose body mass index and atherogenic index of plasma with the risk of diabetes: a retrospective cohort study
Source: Front Endocrinol (Lausanne). 2026 Apr 17;17:1812819. doi: 10.3389/fendo.2026.1812819 (PMC13132742; doi:10.3389/fendo.2026.1812819)
Supplement: Supplementary file 1 [file DataSheet1.docx]

**Supplementary Materials**

Linear associations of triglyceride-glucose body mass index and atherogenic index of plasma with the risk of diabetes: a retrospective cohort study

Sha Wang^1†^, Kui Li^1†^, Xi Wang^1^, Lingjun Zhou^1^, Chancui Deng^1^*, Guanxue Xu^2^*

**Supplementary table 1. Demographic and clinical baseline data for the three groups**

|  | **Total** | **TT1 (TyG-BMI index≤177.48)** | **TT2 (177.48 <** **TyG-BMI index≤211.25)** | **TT3 (TyG-BMI index >** **211.25)** |  |
| --- | --- | --- | --- | --- | --- |
|  | **N=116,662** | **N=38,887** | **N=38,887** | **N=38,888** | ***p*** |
| Age (years) | 41(34,53) | 36(31,45) | 43(34,54) | 46(37,58) | <0.001 |
| Male | 62,759(53.8) | 11,746(30.2) | 21,955(56.5) | 29,058(74.7) | <0.001 |
| BMI (kg/m^2^) | 23.10(21.00,25.40) | 20.10(19.00,21.10) | 23.10(22.20,24.10) | 26.40(25.10,28.00) | <0.001 |
| SBP (mmHg) | 118(107,130) | 110(102,120) | 118(108,129) | 126(115,137) | <0.001 |
| DBP (mmHg) | 73(66,81) | 69(63,76) | 73(67,81) | 79(72,86) | <0.001 |
| Smoking |  |  |  |  | <0.001 |
| Current | 6,658(5.7) | 1,088(2.8) | 2,028(5.2) | 3,542(9.1) |  |
| Former | 1,326(1.1) | 206(0.5) | 465(1.2) | 655(1.7) |  |
| Never | 24,649(21.1) | 8,492(21.8) | 8,265(21.3) | 7,892(20.3) |  |
| Not recorded | 84,029(72.0) | 29,101(74.8) | 28,129(72.3) | 26,799(68.9) |  |
| Drinking |  |  |  |  | <0.001 |
| Current | 872(0.7) | 103(0.3) | 245(0.6) | 524(1.3) |  |
| Former | 5,524(4.7) | 965(2.5) | 1,881(4.8) | 2,678(6.9) |  |
| Never | 26,237(22.5) | 8,718(22.4) | 8,632(22.2) | 8,887(22.9) |  |
| Not recorded | 84,029(72.0) | 29,101(74.8) | 28,129(72.3) | 26,799(68.9) |  |
| Family history | 2,634(2.3) | 861(2.2) | 891(2.3) | 882(2.3) | 0.759 |
| TG (mmol/L) | 1.10(0.76,1.66) | 0.72(0.56,0.94) | 1.10(0.85,1.46) | 1.80(1.33,2.51) | <0.001 |
| TC (mmol/L) | 4.70(4.16,5.32) | 4.41(3.93,4.97) | 4.72(4.20,5.31) | 5.00(4.43,5.62) | <0.001 |
| HDL-C (mmol/L) | 1.35(1.16,1.56) | 1.48(1.29,1.68) | 1.34(1.17,1.54) | 1.24(1.06,1.43) | <0.001 |
| LDL-C (mmol/L) | 2.70(2.29,3.16) | 2.49(2.15,2.90) | 2.75(2.34,3.20) | 2.89(2.45,3.35) | <0.001 |
| FBG (mmol/L) | 4.9(4.6,5.3) | 4.8(4.4,5.1) | 4.9(4.6,5.3) | 5.1(4.8,5.6) | <0.001 |
| ALT(U/L) | 18(13,28) | 14(11,18) | 18(14,25) | 26(19,39) | <0.001 |
| AST(U/L) | 22(19,27) | 20(17,23) | 22(19,26) | 25(21,31) | <0.001 |
| BUN(mmol/L) | 4.570(3.84,5.40) | 4.33(3.64,5.13) | 4.60(3.88,5.41) | 4.76(4.05,5.57) | <0.001 |
| Scr (µmol/L) | 70(58,81) | 62(54,73) | 71(59,82) | 76(65,85) | <0.001 |
| eGFR (mL/min/1.73 m^2^) | 102(90,115) | 107 (95,121) | 101(89,114) | 98(86,111) | <0.001 |
| AIP | 0.27(0.08,0.49) | 0.05(-0.08,0.18) | 0.27(0.14,0.42) | 0.53(0.37,0.70) | <0.001 |

Data are presented as mean±SDs, medians (interquartile ranges), or n (%).

TyG-BMI index tertiles:TT1, TyG-BMI ≤177.48; TT2, 177.48 < TyG-BMI ≤211.25; TT3, TyG-BMI >211.25

Abbreviations: AIP,atherogenic index of plasma;ALT, alanine aminotransferase; AST, aspartate aminotransferase;BMI, body mass index;BUN, blood urea nitrogen;Cr, serum creatinine;DBP, Diastolic Blood Pressure;eGFR, estimated glomerular filtration rate; FBG, fasting blood glucose; HDL-C, high-density lipoprotein cholesterol;LDL-C, low-density lipoprotein cholesterol;SBP, systolic blood pressure;TC, total cholesterol;TG, triglyceride;TyG-BMI index, triglyceride glucose body mass index

**Supplementary table 2. Demographic and clinical baseline data for the three groups**

|  | **Total** | **TA1 (AIP≤0.14)** | **TA2 (0.14<** **AIP≤0.41)** | **TA3 (AIP >** **0.41)** |  |
| --- | --- | --- | --- | --- | --- |
|  | **N=116,662** | **N=38891** | **N=38,887** | **N=38884** | ***p*** |
| Age (years) | 41(34,53) | 37(32,47) | 41(34,53) | 45(36,57) | <0.001 |
| Male | 62,759(53.8) | 12,084(31.1) | 21,563(55.5) | 29,112(74.9) | <0.001 |
| BMI (kg/m^2^) | 23.10(21.00,25.40) | 21.30(19.60,23.20) | 23.10(21.10,25.20) | 25.00(23.10,27.00) | <0.001 |
| SBP (mmHg) | 118(107,130) | 112(103,123) | 118(108,130) | 123(113,135) | <0.001 |
| DBP (mmHg) | 73(66,81) | 70(64,77) | 73(67,81) | 77(70,85) | <0.001 |
| Smoking |  |  |  |  | <0.001 |
| Current | 6,658(5.7) | 910(2.3) | 2,013(5.2) | 3,735(9.6) |  |
| Former | 1,326(1.1) | 240(0.6) | 450(1.2) | 636(1.6) |  |
| Never | 24,649(21.1) | 8,277(21.3) | 8,370(21.5) | 8,002(20.6) |  |
| Not recorded | 84,029(72.0) | 29,464(75.8) | 28,054(72.1) | 26,511(68.2) |  |
| Drinking |  |  |  |  | <0.001 |
| Current | 872(0.7) | 139(0.4) | 266(0.7) | 467(1.2) |  |
| Former | 5,524(4.7) | 1,113(2.9) | 1,857(4.8) | 2,554(6.6) |  |
| Never | 26,237(22.5) | 8,175(21.0) | 8,710(22.4) | 9,352(24.1) |  |
| Not recorded | 84,029(72.0) | 29,464(75.8) | 28,054(72.1) | 26,511(68.2) |  |
| Family history | 2,634(2.3) | 861(2.2) | 860(2.2) | 913(2.3) | 0.341 |
| TG (mmol/L) | 1.10(0.76,1.66) | 0.66(0.53,0.79) | 1.10(0.96,1.29) | 2.00(1.62,2.60) | <0.001 |
| TC (mmol/L) | 4.70(4.16,5.32) | 4.49(4.00,5.03) | 4.69(4.12,5.28) | 5.00(4.40,5.62) | <0.001 |
| HDL-C (mmol/L) | 1.35(1.16,1.56) | 1.55(1.37,1.74) | 1.35(1.21,1.52) | 1.16(1.01,1.32) | <0.001 |
| LDL-C (mmol/L) | 2.70(2.29,3.16) | 2.52(2.17,2.93) | 2.73(2.32,3.18) | 2.87(2.44,3.36) | <0.001 |
| FBG (mmol/L) | 4.9(4.6,5.3) | 4.8(4.5,5.2) | 4.9(4.6,5.3) | 5.1(4.7,5.5) | <0.001 |
| ALT(U/L) | 18(13,28) | 14(11,20) | 18(13,26) | 25(18,37) | <0.001 |
| AST(U/L) | 22(19,27) | 20(17,24) | 22(19,26) | 24 (21,30) | <0.001 |
| BUN(mmol/L) | 4.570(3.84,5.40) | 4.46(3.74,5.30) | 4.57(3.84,5.40) | 4.66(3.97,5.46) | <0.001 |
| Scr (µmol/L) | 70(58,81) | 62(54,74) | 70(59,82) | 76(65,85) | <0.001 |
| eGFR (mL/min/1.73 m^2^) | 102(90,115) | 106(93,120) | 101(89,114) | 99(87,111) | <0.001 |
| TyG-BMI index | 193.90(169.21,221.19) | 165.81(151.88,182.26) | 193.31(175.71,212.06) | 225.76(206.47,247.38) |  |

Data are presented as mean±SDs, medians (interquartile ranges), or n (%).

AIP tertiles: TA1, AIP <0.14; TA2, 0.14-0.41; TA3, AIP >0.41

Abbreviations:

AIP, atherogenic index of plasma;ALT, alanine aminotransferase;AST, aspartate aminotransferase;

BMI, body mass index;BUN,blood urea nitrogen;Cr,creatinine;DBP, Diastolic Blood Pressure; eGFR, estimated glomerular filtration rate;FBG, fasting blood glucose;HDL-C, high-density lipoprotein cholesterol;LDL-C, low-density lipoprotein cholesterol;SBP, systolic blood pressure;TC, total cholesterol;TG,triglyceride;TyG-BMI, triglyceride glucose-body mass index;

**Supplementary table 3. Relationship between TyG-BMI index (per 1 SD) and the risk of diabetes**

|  | OR per SD | 95% CI |
| --- | --- | --- |
| TyG-BMI index≥296.292 | 1.36 | 1.15-1.61 |
| TyG-BMI index<296.292 | 1.63 | 1.55-1.72 |

Atherogenic Index of Plasma, AIP;Confidence Interval, CI;Odds Ratio, OR;Standard Deviation, SD;

Triglyceride Glucose-Body Mass Index, TyG-BMI index

**Supplementary table 4. Relationship between AIP (per 1 SD) and the risk of diabetes**

|  | OR per SD | 95% CI |
| --- | --- | --- |
| AIP≥0.677 | 1.07 | 0.99-1.16 |
| AIP<0.677 | 1.36 | 1.27-1.45 |

Atherogenic Index of Plasma , AIP; Confidence Interval, CI;Odds Ratio, OR; Standard Deviation, SD; Triglyceride Glucose-Body Mass Index, TyG-BMI index

**Supplementary table 5.Linear association between triglyceride-glucose body mass index and risk of diabetes in different ages**

| TyG-BMI index | Events/N | Model1 |  |  |  | Model2 |  |  |  |
| --- | --- | --- | --- | --- | --- | --- | --- | --- | --- |
|  |  | OR | 95%CI | *p* |  | OR | 95%CI | *p* | *P* for interaction |
| Age |  |  |  |  |  |  |  |  | <0.001 |
| ≥60 | 1,124/16,896 | 1.02 | 1.02-1.02 | <0.001 |  | 1.01 | 1.01-1.01 | <0.001 |  |
| T1 | 71/2,652 | Reference |  |  |  | Reference |  |  |  |
| T2 | 274/6,199 | 1.68 | 1.29-2.19 | <0.001 |  | 1.26 | 0.95-1.66 | 0.114 |  |
| T3 | 779/8,045 | 3.90 | 3.04-4.99 | <0.001 |  | 1.89 | 1.43-2.48 | <0.001 |  |
| <60 | 1,557/99,766 | 1.03 | 1.02-1.03 | <0.001 |  | 1.01 | 1.01-1.02 | <0.001 |  |
| T1 | 81/36,235 | Reference |  |  |  | Reference |  |  |  |
| T2 | 274/32,688 | 3.77 | 2.94-4.84 | <0.001 |  | 1.91 | 1.48-2.47 | <0.001 |  |
| T3 | 1,202/30,843 | 18.10 | 14.45-22.68 | <0.001 |  | 4.37 | 3.42-5.58 | <0.001 |  |

Confidence Interval, CI; Odds Ratio, OR; Triglyceride Glucose-body mass index,TyG-BMI index

**Supplementary table 6.Linear association between atherogenic index of plasma and risk of diabetes in different ages**

| AIP | Events/N | Model1 |  |  |  | Model2 |  |  |  |
| --- | --- | --- | --- | --- | --- | --- | --- | --- | --- |
|  |  | OR | 95%CI | *p* |  | OR | 95%CI | *p* | *P* for interaction |
| Age |  |  |  |  |  |  |  |  | <0.001 |
| ≥60 | 1,124/16,896 | 3.10 | 2.50-3.84 | <0.001 |  | 1.77 | 1.31-2.37 | <0.001 |  |
| T1 | 147/3,294 | Reference |  |  |  | Reference |  |  |  |
| T2 | 324/6,045 | 1.21 | 0.99-1.48 | 0.059 |  | 1.07 | 0.86-1.34 | 0.527 |  |
| T3 | 653/7,557 | 2.02 | 1.68-2.43 | <0.001 |  | 1.40 | 1.11-1.76 | 0.004 |  |
| <60 | 1,557/99,766 | 12.24 | 10.53-14.22 | <0.001 |  | 3.39 | 2.75-4.19 | <0.001 |  |
| T1 | 147/35,597 | Reference |  |  |  | Reference |  |  |  |
| T2 | 393/32,842 | 2.92 | 2.42-3.53 | <0.001 |  | 1.76 | 1.44-2.16 | <0.001 |  |
| T3 | 1,017/31,327 | 8.09 | 6.80-9.63 | <0.001 |  | 2.80 | 2.29-3.43 | <0.001 |  |

Atherogenic Index of Plasma, AIP;Confidence Interval, CI;Odds Ratio, OR

**Supplementary table 7. Linear association between triglyceride-glucose body mass index and risk of diabetes in different sex**

| TyG-BMI index | Events/N | Model1 |  |  |  | Model2 |  |  |  |
| --- | --- | --- | --- | --- | --- | --- | --- | --- | --- |
|  |  | OR | 95%CI | *p* |  | OR | 95%CI | *p* | *P* for interaction |
| Sex |  |  |  |  |  |  |  |  | 0.149 |
| Male | 1,888/62,759 | 1.02 | 1.02-1.02 | <0.001 |  | 1.01 | 1.01-1.02 | <0.001 |  |
| T1 | 62/11,746 | Reference |  |  |  | Reference |  |  |  |
| T2 | 340/21,955 | 2.96 | 2.26-3.89 | <0.001 |  | 1.80 | 1.35-2.39 | <0.001 |  |
| T3 | 1,486/29,058 | 10.16 | 7.87-13.10 | <0.001 |  | 3.46 | 2.64-4.53 | <0.001 |  |
| Female | 793/53,903 | 1.03 | 1.03-1.03 | <0.001 |  | 1.01 | 1.01-1.01 | <0.001 |  |
| T1 | 90/27,141 | Reference |  |  |  | Reference |  |  |  |
| T2 | 208/16,932 | 3.74 | 2.92-4.79 | <0.001 |  | 1.31 | 1.01-1.71 | 0.044 |  |
| T3 | 495/9,380 | 15.94 | 12.72-19.98 | <0.001 |  | 2.43 | 1.88-3.15 | <0.001 |  |

Confidence Interval, CI;Odds Ratio, OR;Triglyceride Glucose-body mass index, TyG-BMI index

**Supplementary table 8. Linear association between atherogenic index of plasma and risk of diabetes in different sex**

| **AIP** | Events/N | Model1 |  |  |  | Model2 |  |  |  |
| --- | --- | --- | --- | --- | --- | --- | --- | --- | --- |
|  |  | OR | 95%CI | *p* |  | OR | 95%CI | *p* | *P* for interaction |
| Sex |  |  |  |  |  |  |  |  | 0.818 |
| Male | 1,888/62,759 | 5.58 | 4.79-6.50 | <0.001 |  | 2.82 | 2.31-3.44 | <0.001 |  |
| T1 | 160/12,084 | Reference |  |  |  | Reference |  |  |  |
| T2 | 451/21,563 | 1.59 | 1.33-1.91 | <0.001 |  | 1.34 | 1.10-1.63 | 0.004 |  |
| T3 | 1,277/29,112 | 3.42 | 2.90-4.04 | <0.001 |  | 2.11 | 1.73-2.56 | <0.001 |  |
| Female | 793/53,903 | 16.50 | 13.20-20.61 | <0.001 |  | 2.13 | 1.51-3.01 | <0.001 |  |
| T1 | 90/27,141 | Reference |  |  |  | Reference |  |  |  |
| T2 | 208/16,932 | 3.10 | 2.52-3.82 | <0.001 |  | 1.44 | 1.15-1.82 | 0.002 |  |
| T3 | 495/9,830 | 8.34 | 6.85-10.16 | <0.001 |  | 1.78 | 1.38-2.29 | <0.001 |  |

Atherogenic Index of Plasma, AIP;Confidence Interval, CI;Odds Ratio, OR
